# Differences in Prediction May Underlie Language Disorder in Autism

**DOI:** 10.3389/fpsyg.2022.897187

**Published:** 2022-06-09

**Authors:** Susan Ellis Weismer, Jenny R. Saffran

**Affiliations:** ^1^Waisman Center, University of Wisconsin, Madison, WI, United States; ^2^Department of Communication Sciences and Disorders, University of Wisconsin, Madison, WI, United States; ^3^Department of Psychology, University of Wisconsin, Madison, WI, United States

**Keywords:** predictive coding, hyperplasticity, autism, prediction deficits, language processing

## Abstract

Language delay is often one of the first concerns of parents of toddlers with autism spectrum disorder (ASD), and early language abilities predict broader outcomes for children on the autism spectrum. Yet, mechanisms underlying language deficits in autistic children remain underspecified. One prominent component of linguistic behavior is the use of predictions or expectations during learning and processing. Several researcher teams have posited prediction deficit accounts of ASD. The basic assumption of the prediction accounts is that information is processed by making predictions and testing violations against expectations (prediction errors). Flexible (neurotypical) brains attribute differential weights to prediction errors to determine when new learning is appropriate, while autistic individuals are thought to assign disproportionate weight to prediction errors. According to some views, these prediction deficits are hypothesized to lead to higher levels of perceived novelty, resulting in “hyperplasticity” of learning based on the most recent input. In this article, we adopt the perspective that it would be useful to investigate whether language deficits in children with ASD can be attributed to atypical domain-general prediction processes.

## Introduction

Autism spectrum disorder (ASD) represents a serious and growing public health concern. According to the latest Center for Disease Control and Prevention (CDC) estimates, one in 44 children is currently diagnosed with ASD (Maenner et al., 2021). Although the symptoms and level of severity vary across individuals, ASD has considerable impact on individuals, families, and society (Howlin et al., 2004; Ganz, 2007; Kogan et al., 2008; Lawer et al., 2009; Bradford, 2010). While social communication is universally impaired in this population, there is considerable variation in structural language abilities, i.e., lexicon, syntax (Eigsti et al., 2011; Ellis Weismer and Kover, 2015; Koegel et al., 2020). Early language/communication delays are among the initial red flags for ASD (NICHD, 2021) and are often one of the first concerns noted by parents (De Giacomo and Fombonne, 1998; Eigsti et al., 2011). Importantly, early language abilities predict broader outcomes for children with ASD, including response to treatment and cognitive outcomes (Stone and Yoder, 2001; Szatmari et al., 2003; Eigsti et al., 2011). Therefore, language is a critical area to study and further research on the mechanisms underlying language challenges in autistic children is warranted.

In this article, we advocate the study of predictive processing by children with ASD as it applies to language learning. One prominent component of linguistic behavior is the generation and use of predictions or expectations. Language is processed incrementally by both adults and young children. Mismatches between expected and actual input lead to prediction errors, which are hypothesized to drive learning (e.g., Elman, 1990). Differential weights are attributed to prediction errors by neurotypical brains to determine when new learning is appropriate; however, prediction-based accounts of ASD (Sinha et al., 2014; Van de Cruys et al., 2014) suggest that autistic individuals assign disproportionate weight to prediction errors. These differences in prediction are hypothesized to lead to higher levels of perceived novelty, resulting in “hyperplasticity” of learning in ASD—overweighting the most recent input rather than the aggregation of instances (Sinha et al., 2014). On this view, individuals with ASD downweight prior experiences in favor of recent experiences more than neurotypical individuals. These group differences should be especially evident in changing or variable environments, where recent experiences diverge from prior experiences.

Successful prediction requires sensitivity to statistical regularities in the environment. To the extent that challenges for individuals with ASD are related to hyperplasticity, rather than deficits in statistical learning *per se*, we would expect to see group differences emerge in situations where the environment is more variable, but not when the environment remains relatively static. In addition, we would expect any such differences to be both predictive of language abilities and not limited to language learning tasks.

### Predictive Processes and ASD

Predictive coding theory characterizes perceptual and cognitive representations in terms of probabilistic prediction (Rao and Ballard, 1999; Friston, 2005, 2010; Friston and Kiebel, 2009; Spratling, 2016). Hierarchical predictive coding (also referred to as “predictive processing,” Clark, 2018) entails an implicit process involving the bidirectional flow of information in which bottom–up sensory input is compared to top–down predictions. Incorrect predictions produce error signals. The perceived reliability (or precision) of prediction errors determines the weight assigned to each error with respect to updating predictions. High-precision errors will prompt additional processing and learning whereas low-precision errors typically have less influence.

Several research groups have proposed accounts of ASD (Lawson et al., 2014; Sinha et al., 2014; Van de Cruys et al., 2014) that stem from predictive coding theory. Other studies have employed this theoretical framework to examine more specific areas of functioning within ASD (Gonzalez-Gadea et al., 2015; von der Lühe et al., 2016; Lawson et al., 2017), including auditory processing (Gomot and Wicker, 2012). Prediction deficit accounts of ASD have been linked to previous ASD theories including “extreme male brain” theory (Baron-Cohen, 2002) and intense world theory (Markram and Markram, 2010); prediction models have also been proposed for ASD-typical problems in executive function and central coherence. According to this view, the autism hallmark of restricted and repetitive behaviors serves an adaptive function, compensating for deficits in prediction by insistence on sameness and highly predictable routines.

Sinha et al. (2014) advanced the Predictive Impairment in Autism (PIA) hypothesis as a partial account of the ASD phenotype, focused on the primary diagnostic criteria of social communication deficits and repetitive/restricted behaviors. This framework is based on prior research with neurotypical individuals using Markovian models to characterize gaze behavior, dyadic interactions, mother-infant interactions, smile reciprocity, and communicative interactions. One interesting prediction stemming from the PIA hypothesis (Sinha et al., 2014) has specific implications for learning. Dysfunction in prediction renders higher levels of perceived novelty (see Lawson et al., 2017) leading to hyperarousal of the brainstem and basal ganglia (Joshua et al., 2009) which modulate learning (Schultz et al., 1997; Sutton and Barto, 1998), resulting in hyperplasticity. This hypothesized extreme malleability privileges recent input exposure (disproportionately high weights) and jeopardizes aggregation of prior instances. Hyperplasticity would impair accurate estimation of probabilities when the input changes over time—as in many aspects of the natural world, especially the auditory environment (which is temporally fleeting).

Another prediction account of ASD was proposed by Van de Cruys et al. (2014) based on predictive coding theory (Rao and Ballard, 1999; Friston, 2010). They posit that prior cognitive accounts of ASD—executive dysfunction, theory of mind, and weak central coherence—can all be explained by a core deficit in flexibility in processing violations of expectations. The assumption within this framework is that information is processed by making predictions and detecting violations of expectations (prediction errors). This is an iterative process in which prediction errors (bottom–up information) are influenced by top–down predictions that have been shaped by prior prediction errors. Van de Cruys et al. (2014) suggest that disruptions in bottom-up versus top-down flow of information are reflected in two earlier, opposing cognitive theories of ASD—weak central coherence (bottom-up) versus executive dysfunction (top–down). According to Van de Cruys and colleagues, the core deficit in ASD involves “high, inflexible precision of prediction errors.” That is, individuals with ASD assign inflexibly high weights to prediction errors. Predictive coding involves two time scales (Friston, 2010; Dayan, 2012). While predictions are used for processing the immediate environment, prediction errors shape plasticity and learning over a longer time scale. Sometimes prediction errors indicate that the predictive model should be updated, but other times prediction errors should be ignored. Flexible brains attribute differential weights to prediction errors to determine when new learning is appropriate. Van de Cruys and colleagues hypothesize that individuals with ASD assign too much weight to prediction errors (similar to the hyperplasticity in learning hypothesis of Sinha et al., 2014). If unwarranted high precision is assumed for each prediction error, this induces learning for each new event. Consequently, predictions are noisy and lack generalizability. For instance, there is evidence for enhanced perceptual processing of complex acoustic signals such as speech and tones by individuals with ASD (Heaton, 2003; Jarvinen-Pasley et al., 2008) and it has been suggested that generation of overly specific categories of sounds are detrimental to establishing higher order linguistic representations (Bonnel et al., 2010; Crespi, 2013). Another example pertains to difficulties that autistic individuals appear to have with category learning and extracting prototypes from exemplars (Soulières et al., 2011; Gastgeb et al., 2012). In order for categorization to be successful, it is necessary to recognize certain similarities across new instances of input while also ignoring other within-category variation. Routine use of high precision of prediction errors will impede the ability to establish categories (e.g., autistic children’s category of “dog” may only extend to dogs whose perceptual features are very similar to their own large brown German Shepard and not to dogs of different sizes, colors, or breeds). It is important to note that according to the model proposed by Van de Cruys and colleagues, computation of prediction errors themselves is not impaired in ASD. Instead, learning is impaired or “short-circuited” by a default of indiscriminately high precision which leads to loss of flexible attention allocation based on informativeness. This model proposes that autism deficits in central coherence, executive functions, and theory of mind can be conceptualized in terms of a core deficit in the ability to flexibly process violations of expectations and further that stereotyped behaviors and restricted interests are actually a secondary symptom of this core deficit in (overly precise) predictive processing.

These prediction deficit accounts of ASD have prompted a flurry of research with autistic adults and children. A recent systematic review of the empirical evidence for prediction deficits in ASD assessed findings from 47 studies (Cannon et al., 2021). These investigations spanned infancy through adulthood, tested visual, auditory and audiovisual modalities, and utilized behavioral and neural indices of prediction. Due to the wide range of experimental paradigms and types of data collected by these studies, a formal meta-analysis was not attempted. Instead, Cannon et al. (2021) provide a detailed narrative review of findings and summarize key points of each study in a table. Although some studies failed to find differences in certain predictive skills for individuals with ASD compared to neurotypical individuals (e.g., Manning et al., 2017; Van de Cruys et al., 2018), there is considerable evidence for impairments in learning predictive pairings between an antecedent and consequence particularly in the context of low salience predictive features or variability (Amoruso et al., 2019; Greene et al., 2019; Ganglmayer et al., 2020). Further, results revealed differences in low-level predictive processing, as reflected by habituation and perceptual adaptation (e.g., Turi et al., 2016; Millin et al., 2018; Ruiz-Martínez et al., 2020). It should be noted that none of these studies of predictive skills addressed language processing or language learning in individuals with ASD.

### Statistical Learning and ASD

Successful predictions depend upon sensitivity to patterns in the environment (Van de Cruys et al., 2014). This related area of inquiry has received considerable attention. Statistical learning explanations for ASD have been explored using a range of tasks including serial reaction time (Gordon and Stark, 2007; Travers et al., 2010), contextual cueing (Barnes et al., 2008; Kourkoulou et al., 2012; Travers et al., 2013), probabilistic classification learning (Brown et al., 2010), artificial grammar learning (Brown et al., 2010), speech stream segmentation (Mayo and Eigsti, 2012), and observational learning (Roser et al., 2016). In a meta-analysis, Obeid et al. (Obeid et al., 2016) found no evidence for an overall deficit in statistical learning in ASD. However, a study of visual statistical learning in ASD using ERPs revealed heterogeneity in neural indices of visual statistical learning that were associated with nonverbal IQ and adaptive social function (Jeste et al., 2015).

There are mixed results regarding associations between statistical learning and language abilities in ASD (Scott-Van Zeeland et al., 2010; Mayo and Eigsti, 2012; Haebig et al., 2017). In each of these studies, researchers employed a word segmentation task in which the boundaries between words were indicated by low probabilities of co-occurrences between syllables. Mayo and Eigsti (2012) used a behavioral version of this task with school-aged children with high-functioning autism, and found that their performance was not strongly associated with measures of native language attainment; moreover, their performance was indistinguishable from a comparison group. However, a study using a similar task measuring neural activity *via* fMRI suggested that autistic children may be less sensitive to segmentation cues, with some evidence for a correlation between language measures and recruitment of brain regions believed to be relevant for word segmentation (Scott-Van Zeeland et al., 2010). In a more recent behavioral study with school-aged children, Haebig et al. (2017) also observed an association between statistical learning task performance and measures of English language attainment.

It is important to note that to date, studies of statistical learning in ASD have largely included only participants with relatively strong cognitive and language skills (Obeid et al., 2016) due to the task demands inherent in the statistical learning paradigms commonly used with children and adults. Several studies with (presumably) neurotypical infants and children provide evidence for a relationship between statistical learning performance and language skills (Shafto et al., 2012; Kidd and Arciuli, 2016; Lany et al., 2018), consistent with individual differences observed in adults (Daltrozzo et al., 2017; Siegelman et al., 2017). A similar pattern emerges for children and adolescents with developmental language disorder/specific language impairment who evidence weak performance on both linguistic and nonlinguistic statistical learning tasks (Tomblin et al., 2007; Evans et al., 2009; Obeid et al., 2016; Plante et al., 2017). Taken together, the literature suggests that individual differences in statistical learning task performance may be predictive of language outcomes regardless of whether children have ASD or neurotypical development, at least when the patterns to be learned are relatively static and do not elicit hyperplasticity. The few statistical learning studies that have employed changing input distributions tested only neurotypical adults, and revealed primacy effects in learning (Jungé et al., 2007; Gebhart et al., 2009; Karuza et al., 2016). It remains unclear how individuals with ASD learn from materials in which the input is more variable, such that hyperplasticity may come into play.

### Language and Prediction

Predictive coding theory has been applied to language in studies with neurotypical adults and infants (Gagnepain et al., 2012; Ylinen et al., 2016; Zarcone et al., 2016). For example, Gagnepain et al. (2012) used a predictive coding framework to explain spoken word recognition in neurotypical adults. They contrasted two computational models – lexical competition and segment prediction – using novel word training, computational simulation, and neuroimaging (magnetoencephalographic responses). Their findings supported a segment prediction account in which prediction error signals represent the difference between predicted and heard speech sounds. Ylinen et al. (2016) examined word recognition and learning in neurotypical infants within a predictive coding framework. ERPs were recorded while infants heard native language syllables in an oddball paradigm. The data revealed brain responses reflecting predictive inference. There was a significant correlation between parent-reported receptive vocabulary and mismatch response amplitudes reflecting the strength of prediction errors at 12 months.

Viewed more broadly in terms of incremental language processing, there is an abundance of research demonstrating that neurotypical adults (Altmann and Kamide, 1999; DeLong et al., 2005; Levy, 2008; Kleinman et al., 2015) and infants and toddlers (Lew-Williams and Fernald, 2007; Borovsky et al., 2012; Mahr et al., 2015; Reuter et al., 2021) anticipate upcoming speech (at both lexical and sublexical levels). Children’s ability to predict upcoming nouns from verb semantics relates strongly to their vocabulary knowledge (Borovsky et al., 2012; Mani and Huettig, 2012). Moreover, nonlinguistic measures of prediction in infancy are related to vocabulary size. Reuter et al. (2018), using a task based on Romberg and Saffran (2013), found that infants’ ability to adjust their predictive saccades in a visual task was associated with receptive vocabulary. In sum, the literature to date supports the hypothesis that both linguistic and nonlinguistic predictive processes are related to language processing in neurotypical infants and young children. Currently, we do not have evidence indicating that this link involves a causal relationship or to suggest the direction of influence. Further research involving direct manipulations/longitudinal analyses are needed to ascertain if prediction impacts language gains, language skills influence broader predictive processing, or whether there are, in fact, bidirectional influences between predictive processing and language.

Studies have investigated the ability of children and adolescents with ASD to use semantically informative verbs to predict upcoming nouns (Brock et al., 2008; Bavin et al., 2016; Zhou et al., 2019). Overall, these findings suggest that autistic children can employ predictive language processing, which (like for neurotypical children) is positively associated with their language abilities (Brock et al., 2008; Venker et al., 2019). However, it is important to note that most of this research has only included autistic children with language and nonverbal cognitive abilities in the average range, thereby excluding a significant portion of individuals with ASD and limiting generalizability of the findings. One exception is a recent study by our research team which included a broader sample of young children with ASD (Prescott et al., 2022). Our findings indicated that both the ASD and younger, language-matched neurotypical group made use of semantically informative verbs to predict upcoming nouns as evidenced by anticipatory eye movements. That is, the autistic children (3–4 years of age) performed similarly to language-matched controls (who were on average 18 months younger) in terms of efficiency of predictive language processing. However, regression analyses, when controlling for age, revealed that the ASD group displayed a weaker condition effect (informative vs. neutral verbs) than the neurotypical group, similar to prior research (Zhou et al., 2019). In order to actually evaluate prediction-based accounts of ASD, we need to go beyond these types of incremental language processing tasks. We need to use different paradigms (such as violation of expectation paradigms) that allow us to examine claims about hyperplasticity of learning by manipulating the variability of input and to compare predictive processing for both linguistic and nonlinguistic input. Such tasks will allow us to limit the effects of prior language knowledge (e.g., verb semantics) to isolate predictive behavior based on the statistics of the input, comparing static and variable input distributions.

## Discussion

Despite the clear relevance of predictive processes to both language development and to theories of autism, there have been scant attempts to integrate all three areas of study (prediction, language, and ASD). We propose that research addressing this important gap in the literature is warranted. While there are a range of prediction-based theories related to ASD, as noted above, the initial, ongoing attempts by our research team to explore this issue are not designed to adjudicate between them. We follow Kutas et al. (2014, p. 649) who suggest that prediction encompasses any form of cognitive processing involving “the activation of or information about likely upcoming stimuli, prior to their receipt that plays a causal role in stimulus processing.” Our theoretical framework draws on various aspects of the prediction deficit accounts of ASD discussed above. We assume predictive coding is a domain-general process, but we are especially interested in its role in acquiring and using spoken language. Prior research lays the foundation for prediction deficits in ASD at the level of neural signaling to auditory stimuli (e.g., Gomot and Wicker, 2012; Font-Alaminos et al., 2020), but has not investigated actual language processing or learning in ASD. We hypothesize that children with ASD will demonstrate hyperplasticity of learning (Sinha et al., 2014) which is assumed to stem from difficulty with precision weighting of prediction errors (van de Cruys et al., 2014). We concur with the claims that prediction deficit accounts accommodate many prior cognitive models of autism (Sinha et al., 2014; van de Cruys et al., 2014), but are cautious about assuming that this framework can explain the totality of the ASD phenotype.

It could be argued that there is an apparent paradox in our hypothesis that prediction deficits both underlie autism and are related to language deficits, given that only some children with ASD exhibit structural language impairments. We assume that prediction deficits in the face of social impairments result in the social communication deficits (pragmatic language) that are central to the ASD phenotype. With respect to structural language (vocabulary/grammar), not only are individual differences in prediction assumed to influence these language abilities, but we speculate that individual differences in statistical learning among children with ASD (detecting patterns with stable probabilities), paired with prediction deficits (hyperplasticity) characteristic of ASD, may be related to the observed variability in structural language in autistic children. We contend that a prediction deficit account of language deficits in ASD may be a fruitful line of investigation and encourage other autism researchers to join us in assessing this claim.

## Author’s Note

We alternate between person-first and identify-first language in recognition of the terminology debates among different stakeholders.

## Data Availability Statement

The original contributions presented in the study are included in the article/supplementary material, further inquiries can be directed to the corresponding author.

## Author Contributions

SEW and JRS each wrote sections of this paper and wrote portions of the Introduction and Discussion, added substantive edits to the sections drafted by the other person. SEW drafted paragraphs focused on autism spectrum disorders (ASD), predictive processing and ASD, and predictive coding applied to language. JRS drafted paragraphs focused on statistical learning and predictive language processing in neurotypical children. All authors contributed to the article and approved the submitted version.

## Funding

This work was supported by the National Institutes of Health grants NIDCD R01 DC17974 (SEW and JRS, MPIs) and NICHD U54 HD090256 (Waisman Center core grant) contributed to the writing of this paper.

## Conflict of Interest

The authors declare that the research was conducted in the absence of any commercial or financial relationships that could be construed as a potential conflict of interest.

## Publisher’s Note

All claims expressed in this article are solely those of the authors and do not necessarily represent those of their affiliated organizations, or those of the publisher, the editors and the reviewers. Any product that may be evaluated in this article, or claim that may be made by its manufacturer, is not guaranteed or endorsed by the publisher.
